# Admission Electrolyte Abnormalities and Clinical Outcomes in Hospitalized COVID-19 Patients

**DOI:** 10.3390/medicina62050913

**Published:** 2026-05-08

**Authors:** Emine Önder, Aysun Ekinci, Fırat Aşır, Cigdem Mermutluoglu, Erdal Ozbek, Pakize Gamze Erten Bucaktepe, Ismail Yildiz

**Affiliations:** 1 Department of Biochemistry, Diyarbakir Salahaddin Eyyubi State Hospital, Diyarbakir 21100, Türkiye; emeceb_21@hotmail.com; 2 Department of Biochemistry, Faculty of Medicine, Dicle University, Diyarbakir 21280, Türkiye; 3 Department of Histology and Embryology, Faculty of Medicine, Dicle University, Diyarbakir 21280, Türkiye; firatasir@gmail.com; 4 Department of Infectious Diseases and Clinical Microbiology, Faculty of Medicine, Dicle University, Diyarbakir 21280, Türkiye; cigdemmermut@gmail.com; 5 Department Microbiology, Faculty of Medicine, Dicle University, Diyarbakir 21280, Türkiye; erdal.ozbek@dicle.edu.tr; 6 Department of Family Medicine, Faculty of Medicine, Dicle University, Diyarbakir 21280, Türkiye; gamzebucaktepe@gmail.com; 7 Department of Biostatistics, Faculty of Medicine, Dicle University, Diyarbakir 21280, Türkiye; iyildiz21@yahoo.com

**Keywords:** COVID-19, electrolyte imbalance, hypocalcemia, hyponatremia, intensive care, mechanical ventilation, prognosis, mortality

## Abstract

*Background and Objectives:* Electrolyte abnormalities are frequently observed in hospitalized patients with acute infections and may reflect underlying disease severity. This study aimed to investigate the association between baseline electrolyte disturbances and clinical outcomes in patients with COVID-19, with a particular focus on albumin-corrected calcium levels. *Materials and Methods:* This retrospective study included 348 hospitalized patients with COVID-19. Primary analyses were restricted to RT-PCR-confirmed cases (n = 272) to minimize misclassification bias, while the full cohort was evaluated in sensitivity analyses. Baseline electrolyte levels at admission were recorded, and corrected calcium levels were calculated using serum albumin. Clinical outcomes included prolonged hospitalization (defined relative to the cohort median), intensive care unit (ICU) admission, invasive mechanical ventilation (IMV), and in-hospital mortality. Multivariable logistic regression analyses were performed adjusting for age, sex, and renal function (eGFR). *Results*: In the PCR-confirmed cohort, corrected hypocalcemia was present in 37.3% of patients. In univariate analyses, hypocalcemia, hyponatremia, and hypophosphatemia were significantly associated with adverse outcomes. However, after adjustment, corrected hypocalcemia did not retain independent significance. Hyponatremia remained independently associated with ICU admission (OR: 9.45, 95% CI: 2.12–42.1, *p* = 0.003), while hypophosphatemia was independently associated with prolonged hospitalization (OR: 2.83, 95% CI: 1.36–5.91, *p* = 0.005). No electrolyte abnormality demonstrated a stable independent association with IMV requirement or mortality after adjustment. Sensitivity analyses in the full cohort yielded consistent findings. *Conclusions*: Electrolyte abnormalities are common in hospitalized COVID-19 patients and are associated with worse clinical outcomes; however, they primarily reflect overall disease severity rather than acting as independent prognostic determinants. Routine electrolyte measurements may provide accessible and clinically informative markers but should be interpreted in conjunction with other clinical parameters.

## 1. Introduction

Coronaviruses are a family of viruses that can cause disease across a wide clinical spectrum, ranging from upper respiratory tract infections to acute respiratory distress syndrome (ARDS) [1]. Severe Acute Respiratory Syndrome Coronavirus-2 (SARS-CoV-2), which emerged in 2019, has become a significant global public health problem due to its heterogeneous clinical course, ranging from asymptomatic infection to multiple organ failure, and its high transmissibility [2]. The unpredictable clinical course of COVID-19 has made early identification of high-risk patients upon hospital admission and planning of appropriate clinical management critical.

COVID-19 diagnosis is made by Reverse Transcription Polymerase Chain Reaction (RT-PCR) test, which is based on the demonstration of viral nucleic acid and/or clinical and radiological findings [3]. Routine biochemical parameters such as complete blood count (CBC), inflammation markers, and serum electrolytes are commonly used in evaluating the prognosis of the disease [4]. Serum electrolytes are potentially widely available laboratory parameters that may provide early clinical information due to their low cost, rapid results, and measurability in almost all patients admitted to the hospital.

It has been reported that electrolyte imbalances in COVID-19 may develop because of multiple mechanisms, including changes in angiotensin-converting enzyme-2 (ACE2)-mediated renin-angiotensin system activation, systemic inflammatory response, gastrointestinal losses, and renal dysfunction [5,6,7].

Recent studies have more clearly revealed the role of electrolyte disorders in the pathophysiology of COVID-19. Hyponatremia because of syndrome of inappropriate antidiuresis (SIAD) has been reported to be common in COVID-19 and may be closely related to inflammatory processes [8]. Similarly, systematic review findings showing that low serum magnesium levels are associated with increased disease severity highlight the regulatory role of magnesium in the immune response and endothelial function [9]. On the other hand, hypocalcemia has been shown to be associated with adverse clinical outcomes, particularly in COVID-19 patients with diabetes, and it has been emphasized that calcium metabolism may be important in disease prognosis [10]. In addition, it has been reported that magnesium replacement can have ameliorative effects on clinical and biochemical parameters, and this finding suggests that electrolyte imbalances may not only be an indicator of disease but also a potentially modifiable risk factor [11].

However, most existing studies have evaluated individual electrolyte abnormalities in isolation, and comprehensive analyses assessing whether multiple electrolyte disturbances remain associated with adverse outcomes after adjustment for major confounding factors within the same patient cohort remain limited [12,13]. Moreover, although electrolyte imbalances are frequently reported in COVID-19, their integration into multivariable models for early risk stratification has not been consistently addressed [14]. Early recognition of electrolyte abnormalities can contribute to the identification of high-risk patients, helping to plan treatment strategies in a timely manner and optimize clinical management [15,16].

The aim of this study was to evaluate whether baseline electrolyte abnormalities at hospital admission were associated with adverse clinical outcomes in hospitalized COVID-19 patients, and whether these associations remained significant after adjustment for age, sex, and renal function.

## 2. Materials and Methods

### 2.1. Study Design and Population

This retrospective observational study was conducted with the approval of the Dicle University Faculty of Medicine Non-Interventional Clinical Research Ethics Committee and the Scientific Research Platform of the Ministry of Health of the Republic of Turkey (Decision date: 7 November 2022, decision no: 225). Between 1 April 2020 and 1 July 2020, a total of 640 adult patients hospitalized in the COVID-19 ward or intensive care unit (ICU) were screened. Of these, 348 patients (178 men, 170 women) who met the inclusion criteria were included in the analyses. COVID-19 diagnosis was established based on the following criteria:

1. Positive RT-PCR test for SARS-CoV-2, or
2. Negative RT-PCR result but fulfillment of all of the following:
  • Presence of fever and/or respiratory symptoms.
  • Thoracic imaging findings compatible with COVID-19 [17].
  • Presence of lymphopenia with normal or low white blood cell count [18].

Inclusion criteria were:

• Age ≥ 18 years.
• Confirmed or clinically compatible COVID-19 diagnosis.
• Availability of complete laboratory data at admission.

Exclusion criteria were:

• Age < 18 years.
• Pregnancy.
• Incomplete clinical or laboratory data.

The inclusion of RT-PCR-negative but clinically and radiologically compatible patients was intended to reflect real-world clinical practice during the early phase of the pandemic.

### 2.2. Data Collection and Clinical Definitions

Demographic data (age, sex), clinical outcomes, and laboratory parameters at admission were retrospectively obtained from the hospital information management system. Clinical outcomes included:

• ICU admission.
• Length of hospital stay.
• Requirement for invasive mechanical ventilation (IMV).
• In-hospital mortality.

Patients transferred to the ICU during hospitalization due to clinical deterioration were classified as requiring ICU admission. Prolonged hospitalization was defined relative to the median length of stay of the study cohort.

### 2.3. Laboratory Measurements

Laboratory parameters obtained at admission included complete blood count (white blood cells, lymphocytes, platelets), D-dimer, ferritin, C-reactive protein (CRP), fibrinogen, procalcitonin, erythrocyte sedimentation rate (ESR), and serum electrolytes (sodium, potassium, calcium, phosphorus, magnesium, and chloride). CBC parameters were measured using a Sysmex XN-100 (Sysmex Corporation, Kobe, Japan), biochemical parameters using a Beckman Coulter AU 5800 (Beckman Coulter Inc., Brea, CA, USA), ferritin using a Siemens Advia Centaur XP (Siemens Healthcare Diagnostics Inc., Tarrytown, NY, USA), ESR using a Vision-C system (Menarini Diagnostics, Firenze, Italy), fibrinogen using a STA Compact Max® 3 system (Diagnostica Stago, Asnières-sur-Seine, France), and procalcitonin and D-dimer using a Radiometer AQT90 Flex system (Radiometer Medical ApS, Brønshøj, Denmark). RT-PCR analyses were performed using validated commercial kits in accordance with the manufacturers’ instructions. Results were interpreted based on appropriate control validation and cycle threshold values.

### 2.4. Definition of Electrolyte Abnormalities

All laboratory parameters were classified as low, normal, or high according to institutional reference ranges. For the primary analyses, electrolyte variables were dichotomized as low versus normal/high because the number of patients in the high category was small and resulted in unstable estimates. Albumin-corrected calcium levels were calculated and used in the primary analyses to provide a more accurate assessment of calcium status in the presence of potential hypoalbuminemia. Hypocalcemia and hyponatremia were considered primary exposures because they were the most prevalent abnormalities and showed the strongest associations in initial analyses. Other electrolyte abnormalities were evaluated in an exploratory manner.

### 2.5. Statistical Analysis

Statistical analyses were performed using IBM SPSS Statistics for Windows (Version 21.0; IBM Corp., Armonk, NY, USA). Continuous variables were expressed as mean ± standard deviation (SD), while categorical variables were expressed as number and percentage (%). Normality of distribution was assessed using the Shapiro–Wilk and Kolmogorov–Smirnov tests. Comparisons between two independent groups were performed using the independent samples t-test for normally distributed variables and the Mann–Whitney U test for non-normally distributed variables. For comparisons involving more than two groups, one-way ANOVA or Kruskal–Wallis tests were applied as appropriate. Categorical variables were compared using the chi-square test or Fisher’s exact test when appropriate.

Univariate and multivariable logistic regression analyses were performed to evaluate the association between electrolyte abnormalities and clinical outcomes. Multivariable models were adjusted for age, sex, and renal function (eGFR), which were considered clinically relevant confounders. Electrolyte variables considered clinically relevant and/or showing significant associations in univariate analyses were included in the multivariable models. Given the limited number of events for some outcomes, the number of predictors included in multivariable models was restricted in accordance with events-per-variable considerations. For outcomes with sparse events (IMV requirement and mortality), parsimonious models were applied. Due to the retrospective design, detailed data on comorbidities and treatment-related variables were not consistently available and could not be included in the models. All statistical tests were two-sided, and a *p*-value < 0.05 was considered statistically significant.

## 3. Results

### 3.1. Baseline Characteristics

A total of 348 patients were initially included in the study. To minimize misclassification bias, primary analyses were restricted to 272 patients with RT-PCR-confirmed COVID-19. The mean age of the PCR-positive cohort was 45.8 ± 17.9 years, and 50.7% of the patients were male. The median hospital stay was 5 days (IQR: 3–7). ICU admission was required in 10.3% of patients, while 6.3% required invasive mechanical ventilation (IMV) and 6.3% died during hospitalization. At admission, the most common electrolyte abnormalities were hyponatremia (41.2%), corrected hypocalcemia (37.3%), hypomagnesemia (30.1%), and hypophosphatemia (17.3%). Baseline laboratory characteristics of the study population are summarized in Table 1.

**Figure 1:**
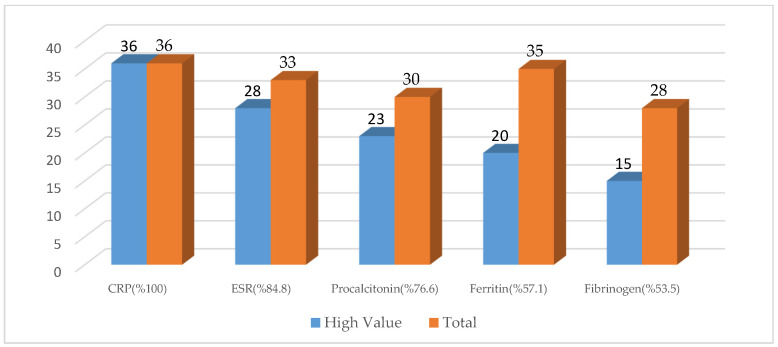
Levels of inflammatory markers in ICU patients.

In patients admitted to the intensive care unit, CRP was found to be elevated in 100%, ESR in 84.8%, procalcitonin in 76.6%, ferritin in 57.1%, and fibrinogen in 53.5% (Figure 1).

### 3.2. Association Between Electrolyte Abnormalities and Clinical Outcomes

Hyponatremia, corrected hypocalcemia, and hypophosphatemia were significantly associated with ICU admission in univariate analyses (*p* < 0.05). In contrast, hypomagnesemia did not show a statistically significant association with ICU requirement (Table 2). Similarly, hyponatremia and hypophosphatemia were associated with prolonged hospital stay (*p* < 0.05), whereas corrected hypocalcemia showed only a borderline association (Table 3). For IMV requirement and in-hospital mortality, electrolyte abnormalities demonstrated significant associations in univariate analyses; however, these findings were based on a limited number of events and were interpreted cautiously.

**Table 2:** Association between baseline electrolyte abnormalities and ICU admission (PCR-positive cohort).

Variable	No ICU, n (%)	ICU, n (%)	*p* Value
Hyponatremia	100 (89.3)	12 (10.7)	<0.001
Hypocalcemia (corrected)	86 (85.1)	15 (14.9)	0.012
Hypophosphatemia	38 (80.4)	9 (19.6)	0.021
Hypomagnesemia	74 (90.2)	8 (9.8)	0.215

Values are presented as number (percentage) within each group. The chi-square test was used for comparisons; Fisher’s exact test was used when appropriate.

### 3.3. Multivariable Analysis

Multivariable logistic regression analyses were performed to evaluate the independent association between electrolyte abnormalities and clinical outcomes, adjusting for age, sex, and renal function (eGFR). Hyponatremia remained a strong independent predictor of ICU admission (OR: 9.45, 95% CI: 2.12–42.1, *p* = 0.003). Corrected hypocalcemia showed a borderline association (OR: 3.20, 95% CI: 0.92–11.08, *p* = 0.070), whereas hypophosphatemia was not independently associated with ICU requirement. Increasing age (OR: 1.06, *p* = 0.002) and lower eGFR (OR: 0.98, *p* = 0.011) were also independently associated with ICU admission. Hypophosphatemia remained independently associated with prolonged hospital stay (OR: 2.83, 95% CI: 1.36–5.91, *p* = 0.005). In contrast, hyponatremia and corrected hypocalcemia were not independently associated with this outcome after adjustment. Age (OR: 1.03, *p* = 0.001) and eGFR (OR: 0.99, *p* = 0.041) were also significantly associated with longer hospitalization. Due to the limited number of events, parsimonious models were used for IMV requirement and in-hospital mortality. In these analyses, electrolyte abnormalities, including corrected hypocalcemia and hyponatremia, were not independently associated with IMV requirement or mortality. However, increasing age and lower eGFR remained significant predictors of both outcomes (Table 4). 

### 3.4. Sensitivity Analysis

Sensitivity analyses performed in the full cohort (including RT-PCR-negative but clinically diagnosed patients) demonstrated similar overall patterns, particularly regarding the association of hyponatremia with ICU admission and hypophosphatemia with prolonged hospitalization. 

Supplementary Tables S1–S3 present detailed analyses performed in the full cohort (n = 348), including baseline characteristics, univariate analyses, and multivariable regression models. These analyses were conducted to assess the robustness of the findings obtained in the RT-PCR-confirmed cohort.

## 4. Discussion

Since the onset of the COVID-19 pandemic, identifying reliable, accessible, and clinically meaningful predictors of disease severity has remained a central objective in hospital medicine. In this context, routine biochemical parameters, particularly electrolyte measurements, have attracted attention due to their widespread availability and low cost [19,20]. The present study demonstrates that baseline electrolyte abnormalities are common in hospitalized patients with COVID-19 and are associated with adverse clinical outcomes. However, after adjustment for key clinical factors, these abnormalities appear to primarily reflect overall disease severity rather than acting as independent predictors of hard clinical outcomes.

Importantly, the use of albumin-corrected calcium in the present study provides a more physiologically accurate assessment of calcium status. This methodological approach may partly explain the attenuation of the association between hypocalcemia and clinical outcomes in adjusted analyses [21]. In contrast to several previous reports that relied on total calcium levels, our findings suggest that uncorrected calcium measurements may overestimate the prognostic significance of hypocalcemia in inflammatory conditions such as COVID-19 [22,23].

Although electrolyte measurements are widely available and may provide early clinical signals, their incremental value for risk stratification beyond established clinical predictors remains uncertain [24]. Therefore, electrolyte abnormalities should be interpreted in conjunction with clinical and physiological parameters rather than as standalone prognostic tools. This distinction is particularly relevant in the context of multivariable analyses, where most electrolyte disturbances did not retain independent associations with outcomes such as mechanical ventilation or mortality [25,26].

Hyponatremia was one of the most prevalent electrolyte abnormalities in our cohort and demonstrated a strong and independent association with ICU admission in multivariable analyses restricted to RT-PCR-confirmed patients [27,28]. This finding is consistent with previous studies suggesting that hyponatremia may reflect disease severity through mechanisms such as syndrome of inappropriate antidiuretic hormone secretion (SIADH), inflammatory cytokine activation, and pulmonary involvement [29,30,31]. The strong gradient observed between hyponatremia and adverse outcomes further supports its role as a clinically relevant marker of a severe disease phenotype [32,33,34].

In contrast, corrected hypocalcemia did not demonstrate a consistent independent association with clinical outcomes after adjustment. This finding suggests that previously reported associations between hypocalcemia and poor prognosis may, at least in part, be influenced by uncorrected calcium measurements and underlying hypoalbuminemia. These results highlight the importance of using corrected or ionized calcium levels when evaluating calcium-related prognostic associations, particularly in patients with systemic inflammation and altered protein metabolism [21,35].

Phosphate disturbances have been increasingly recognized in critically ill patients and are often associated with metabolic stress, respiratory dysfunction, and prolonged hospitalization [36,37,38]. In the present study, hypophosphatemia remained independently associated with prolonged hospital stay after adjustment, supporting its potential role as a marker of disease burden and metabolic derangement. This finding is consistent with previous reports linking phosphate abnormalities to adverse clinical outcomes in critically ill populations, including patients with COVID-19 [38,39].

Electrolyte abnormalities involving magnesium and chloride demonstrated associations with clinical outcomes in univariate analyses; however, these associations were not consistently maintained after adjustment. This pattern suggests that such abnormalities may reflect secondary effects of critical illness, including renal dysfunction, fluid shifts, and treatment-related factors, rather than serving as independent drivers of disease progression [40,41,42].

An important finding of the present study is that, when key confounders such as age and renal function are considered, most electrolyte abnormalities lose their independent predictive value for hard clinical outcomes such as mechanical ventilation and mortality. This supports the concept that electrolyte disturbances function as integrative biomarkers of systemic dysfunction rather than causal determinants of disease progression [43,44]. In this context, they may still provide clinically meaningful information, particularly when interpreted alongside other laboratory and physiological parameters.

Although the study population reflects the early phase of the COVID-19 pandemic, the clinical implications of our findings are likely to extend beyond SARS-CoV-2 infection. Electrolyte disturbances represent fundamental physiological responses to systemic inflammation, critical illness, and organ dysfunction, and therefore may retain relevance across a broad spectrum of acute medical conditions [45,46].

The present study has several limitations that should be acknowledged. First, its retrospective design precludes causal inference between electrolyte abnormalities and clinical outcomes. In addition, the etiology of electrolyte disturbances could not be evaluated in detail because data regarding medication use (particularly diuretics), fluid therapy, syndrome of inappropriate antidiuretic hormone secretion (SIADH), gastrointestinal and renal losses, and nutritional status were not consistently available. The absence of serial electrolyte measurements further limited the ability to assess the prognostic significance of dynamic changes during hospitalization. Although albumin data were available for a subset of patients and albumin-corrected calcium was used in the primary analyses, ionized calcium measurements were not available. Therefore, misclassification related to calcium status cannot be completely excluded, particularly in patients with significant inflammatory burden or altered protein metabolism.

Another important limitation is the relatively small number of events for some outcomes, particularly invasive mechanical ventilation (IMV) requirement and mortality, which may have reduced statistical power and increased the risk of unstable estimates in multivariable models. To address this issue, parsimonious modeling strategies were applied; however, these results should still be interpreted with caution. The inclusion of RT-PCR-negative but clinically and radiologically compatible patients reflects real-world practice during the early pandemic period, when RT-PCR sensitivity and availability were limited. However, this approach may have introduced heterogeneity and potential misclassification bias. To mitigate this, primary analyses were restricted to RT-PCR-confirmed cases, and sensitivity analyses demonstrated consistent overall patterns, supporting the robustness of the main findings.

Finally, residual confounding remains possible because detailed data on comorbidities and treatment-related factors were not consistently available. As a result, electrolyte abnormalities identified in this study should be interpreted primarily as markers of disease severity rather than independent causal determinants. Future multicenter, prospective studies incorporating comprehensive clinical data and serial laboratory measurements are needed to clarify whether correction of electrolyte disturbances can improve clinical outcomes.

## 5. Conclusions

Our findings suggest that electrolyte abnormalities should not be regarded merely as laboratory irregularities, but rather as clinically relevant markers associated with disease severity and adverse outcomes in hospitalized patients with COVID-19. Among the evaluated electrolyte abnormalities, hypocalcemia and hyponatremia were associated particularly with ICU admission, whereas hypophosphatemia was associated with prolonged hospitalization. However, no electrolyte abnormality demonstrated a stable independent association with IMV requirement or mortality after adjustment for age, sex, and renal function. These findings support the use of routine electrolyte measurements as practical, accessible, and cost-effective adjunctive markers that may support clinical assessment in hospitalized patients. Nevertheless, because of the retrospective design, potential residual confounding, and limited number of events for some outcomes, the results should be interpreted cautiously. Prospective multicenter studies are needed to further clarify the prognostic value of electrolyte disturbances and determine whether correction of these abnormalities can improve clinical outcomes.

## Figures and Tables

**Table 1:** Baseline characteristics of RT-PCR-confirmed patients (n = 272).

Demographic and Clinical Characteristics			
Variable	Mean ± SD	Median (IQR)	Min–Max
Age (years)	45.8 ± 17.9	44 (32–58)	18–91
Hospital stay (days)	5.42 ± 3.98	5 (3–7)	1–28
eGFR (mL/min/1.73 m^2^)	106.4 ± 28.9	109 (92–124)	6–185
Variable	n (%)		
Male sex	138 (50.7)		
ICU admission	28 (10.3)		
IMV requirement	17 (6.3)		
In-hospital mortality	17 (6.3)		
Laboratory Parameters at Admission			
Variable	Mean ± SD	Median (IQR)	Min–Max
Sodium (mmol/L)	136.5 ± 3.3	136 (134–139)	122–160
Potassium (mmol/L)	4.17 ± 0.49	4.10 (3.90–4.40)	2.8–7.1
Chloride (mmol/L)	103.6 ± 3.4	104 (101–106)	90–117
Corrected calcium (mg/dL)	8.92 ± 0.62	8.90 (8.50–9.30)	7.2–10.6
Magnesium (mg/dL)	2.04 ± 0.28	2.00 (1.85–2.20)	1.2–3.3
Phosphate (mg/dL)	3.14 ± 0.74	3.10 (2.70–3.60)	1.3–7.1
CRP (mg/dL)	3.08 ± 5.10	1.20 (0.40–3.90)	0.02–32.9
Ferritin (ng/mL)	245.3 ± 351.7	120 (45–310)	2–3700
D-dimer (mg/L)	1.70 ± 6.20	0.40 (0.20–0.90)	0.08–96
WBC (10^3^/µL)	6.85 ± 3.20	6.10 (4.80–7.90)	2.4–28
Lymphocytes (10^3^/µL)	1.70 ± 0.72	1.60 (1.10–2.10)	0.3–4.8
Platelets (10^3^/µL)	232.8 ± 82.9	220 (180–270)	22–690
Fibrinogen (mg/dL)	320.5 ± 125.4	310 (240–390)	86–760
Prevalence of Electrolyte Abnormalities			
Variable	n (%)		
Hyponatremia	112 (41.2)		
Hypocalcemia (corrected)	101 (37.3)		
Hypophosphatemia	47 (17.3)		
Hypomagnesemia	82 (30.1)		

Continuous variables are presented as mean ± standard deviation (SD) and median (interquartile range, IQR). Categorical variables are presented as number (percentage). eGFR: estimated glomerular filtration rate; ICU: intensive care unit; IMV: invasive mechanical ventilation.

**Table 3:** Association between electrolyte abnormalities and hospital length of stay (>median).

Variable	≤Median Stay, n (%)	>Median Stay, n (%)	*p* Value
Hyponatremia	72 (64.1)	40 (35.9)	0.018
Hypocalcemia (corrected)	61 (60.3)	40 (39.7)	0.062
Hypophosphatemia	23 (48.9)	24 (51.1)	<0.001
Hypomagnesemia	52 (63.5)	30 (36.5)	0.210

Prolonged hospitalization was defined relative to the cohort median length of stay. Values are presented as number (percentage). The chi-square test was used for comparisons.

**Table 4:** Multivariable logistic regression analysis (PCR-positive cohort).

ICU Admission			
Variable	OR	95% CI	*p* Value
Hyponatremia	9.45	2.12–42.1	0.003
Hypocalcemia (corrected)	3.20	0.92–11.08	0.070
Hypophosphatemia	1.88	0.61–5.79	0.272
Age	1.06	1.02–1.10	0.002
eGFR	0.98	0.96–0.99	0.011
Prolonged Hospitalization			
Variable	OR	95% CI	*p* Value
Hypophosphatemia	2.83	1.36–5.91	0.005
Hyponatremia	1.42	0.79–2.55	0.240
Hypocalcemia (corrected)	1.31	0.72–2.39	0.371
Age	1.03	1.01–1.05	0.001
eGFR	0.99	0.98–1.00	0.041
IMV & Mortality (parsimonious model)			
Variable	OR	95% CI	*p* Value
Hypocalcemia (corrected)	2.11	0.58–7.65	0.258
Hyponatremia	1.98	0.52–7.43	0.314
Age	1.05	1.01–1.10	0.018
eGFR	0.96	0.93–0.99	0.009

OR: odds ratio; CI: confidence interval. Multivariable logistic regression models were adjusted for age, sex, and eGFR. For outcomes with limited events (IMV and mortality), parsimonious models were applied.

## Data Availability

The original contributions presented in this study are included in the article/Supplementary Material. Further inquiries can be directed to the corresponding author.

## Supplementary Materials

The following supporting information can be downloaded at: https://www.mdpi.com/article/10.3390/medicina62050913/s1, Supplementary Tables S1–S3 present detailed analyses performed in the full cohort (n = 348), including baseline characteristics, univariate analyses, and multivariable regression models. These analyses were conducted to assess the robustness of the findings obtained in the RT-PCR-confirmed cohort.

## Abbreviations

ACE2	angiotensin-converting enzyme 2;
ARDS	acute respiratory distress syndrome
CBC	complete blood count
CI	confidence interval
CRP	C-reactive protein
CT	cycle threshold
eGFR	estimated glomerular filtration rate
ESR	erythrocyte sedimentation rate
HIMS	hospital information management system
ICU	intensive care unit
IL-6	interleukin-6
IMV	invasive mechanical ventilation
OR	odds ratio
qPCR	quantitative polymerase chain reaction
RAS	renin–angiotensin system
RT-PCR	reverse transcription polymerase chain reaction
SD	standard deviation
SIADH	syndrome of inappropriate antidiuretic hormone secretion
SARS-CoV-2	severe acute respiratory syndrome coronavirus 2
WBC	white blood cell
