# Supplementary material for: Admission Electrolyte Abnormalities and Clinical Outcomes in Hospitalized COVID-19 Patients
Source: Medicina (Kaunas). 2026 May 8;62(5):913. doi: 10.3390/medicina62050913 (PMC13208330; doi:10.3390/medicina62050913)
Supplement: Supplementary file 1 [file medicina-62-00913-s001.zip › medicina-4255894-supplementary.pdf]

Supplementary Tables

Table S1. Baseline characteristics of the full cohort (n = 348)

Variable	Mean ± SD	Median (IQR)	Min–Max	n (%)
Age (years)	46.2 ± 18.3	45 (33–59)	18–93	—
Male sex	—	—	—	178 (51.1)
Hospital stay (days)	5.61 ± 4.12	5 (3–7)	1–32	—
eGFR (mL/min/1.73 m²)	104.8 ± 31.2	107 (90–123)	5–190	—
ICU admission	—	—	—	36 (10.3)
IMV requirement	—	—	—	22 (6.3)
In-hospital mortality	—	—	—	20 (5.7)
Sodium (mmol/L)	136.4 ± 3.5	136 (134–139)	121–161	—
Potassium (mmol/L)	4.16 ± 0.52	4.10 (3.90–4.40)	2.7–7.3	—
Chloride (mmol/L)	103.5 ± 3.6	104 (101–106)	89–118	—
Corrected calcium (mg/dL)	8.90 ± 0.65	8.88 (8.50–9.30)	7.0–10.8	—
Magnesium (mg/dL)	2.03 ± 0.30	2.00 (1.84–2.20)	1.1–3.5	—
Phosphate (mg/dL)	3.12 ± 0.79	3.10 (2.70–3.60)	1.2–7.4	—
Hyponatremia	—	—	—	146 (42.0)
Hypocalcemia (corrected)	—	—	—	132 (37.9)
Hypophosphatemia	—	—	—	60 (17.2)
Hypomagnesemia	—	—	—	105 (30.2)

Table S2. Univariate analysis in the full cohort

Univariate analysis in the full cohort			
**Variable**	**No ICU (%)**	**ICU (%)**	**p value**
Hyponatremia	88.5	11.5	<0.001
Hypocalcemia (corrected)	84.2	15.8	0.008
Hypophosphatemia	78.0	22.0	0.015
Hypomagnesemia	89.1	10.9	0.198
**Prolonged hospitalization (> median)**			
**Variable**	**≤ median (%)**	**> median (%)**	**p value**
Hyponatremia	63.0	37.0	0.021
Hypocalcemia (corrected)	59.4	40.6	0.070
Hypophosphatemia	47.5	52.5	<0.001
Hypomagnesemia	62.1	37.9	0.233

Table S3. Multivariable logistic regression analysis in the full cohort

ICU Admission			
**Variable**	**OR**	**95% CI**	**p value**
Hyponatremia	8.72	2.05–37.1	0.004
Hypocalcemia (corrected)	2.95	0.88–9.86	0.081
Hypophosphatemia	1.76	0.59–5.20	0.309
Age	1.05	1.02–1.09	0.003
eGFR	0.98	0.96–0.99	0.014
**Prolonged Hospitalization**			
**Variable**	**OR**	**95% CI**	**p value**
Hypophosphatemia	2.67	1.30–5.45	0.007
Hyponatremia	1.39	0.80–2.41	0.245
Hypocalcemia (corrected)	1.28	0.73–2.25	0.381
Age	1.03	1.01–1.05	0.001
eGFR	0.99	0.98–1.00	0.046
**IMV & Mortality (parsimonious)**			
**Variable**	**OR**	**95% CI**	**p value**
Hypocalcemia (corrected)	2.03	0.60–6.88	0.257
Hyponatremia	1.91	0.54–6.72	0.313
Age	1.05	1.01–1.09	0.019
eGFR	0.96	0.93–0.99	0.011
